# Supplementary material for: Hybridized quantum dot, silica, and gold nanoparticles for targeted chemo-radiotherapy in colorectal cancer theranostics
Source: Commun Biol. 2024 Apr 1;7:393. doi: 10.1038/s42003-024-06043-6 (PMC10984983; doi:10.1038/s42003-024-06043-6)
Supplement: Supplementary file 2 — Supplementary Information.pdf [file 42003_2024_6043_MOESM2_ESM.docx]

**Hybridized quantum dot, silica, and gold nanoparticles for targeted chemo-radiotherapy in colorectal cancer theranostics**

**Amir Abrishami ^1^, Ahmad Reza Bahrami ^1,2^,** **Sirous Nekooei ^3^, Amir Sh. Saljooghi ^4,5*^ and Maryam M. Matin ^1,5*^**

^1^ Department of Biology, Faculty of Science, Ferdowsi University of Mashhad, Mashhad, Iran

^2^ Industrial Biotechnology Research Group, Institute of Biotechnology, Ferdowsi University of Mashhad, Mashhad, Iran

^3^ Department of Radiology, Faculty of Medicine, Mashhad University of Medical Sciences, Mashhad, Iran

^4^ Department of Chemistry, Faculty of Science, Ferdowsi University of Mashhad, Mashhad, Iran

^5^ Novel Diagnostics and Therapeutics Research Group, Institute of Biotechnology, Ferdowsi University of Mashhad, Mashhad, Iran

^*^ Corresponding Authors’ e-mails: saljooghi@um.ac.ir; [matin@um.ac.ir](mailto:matin@um.ac.ir)

**Supplementary Figures**


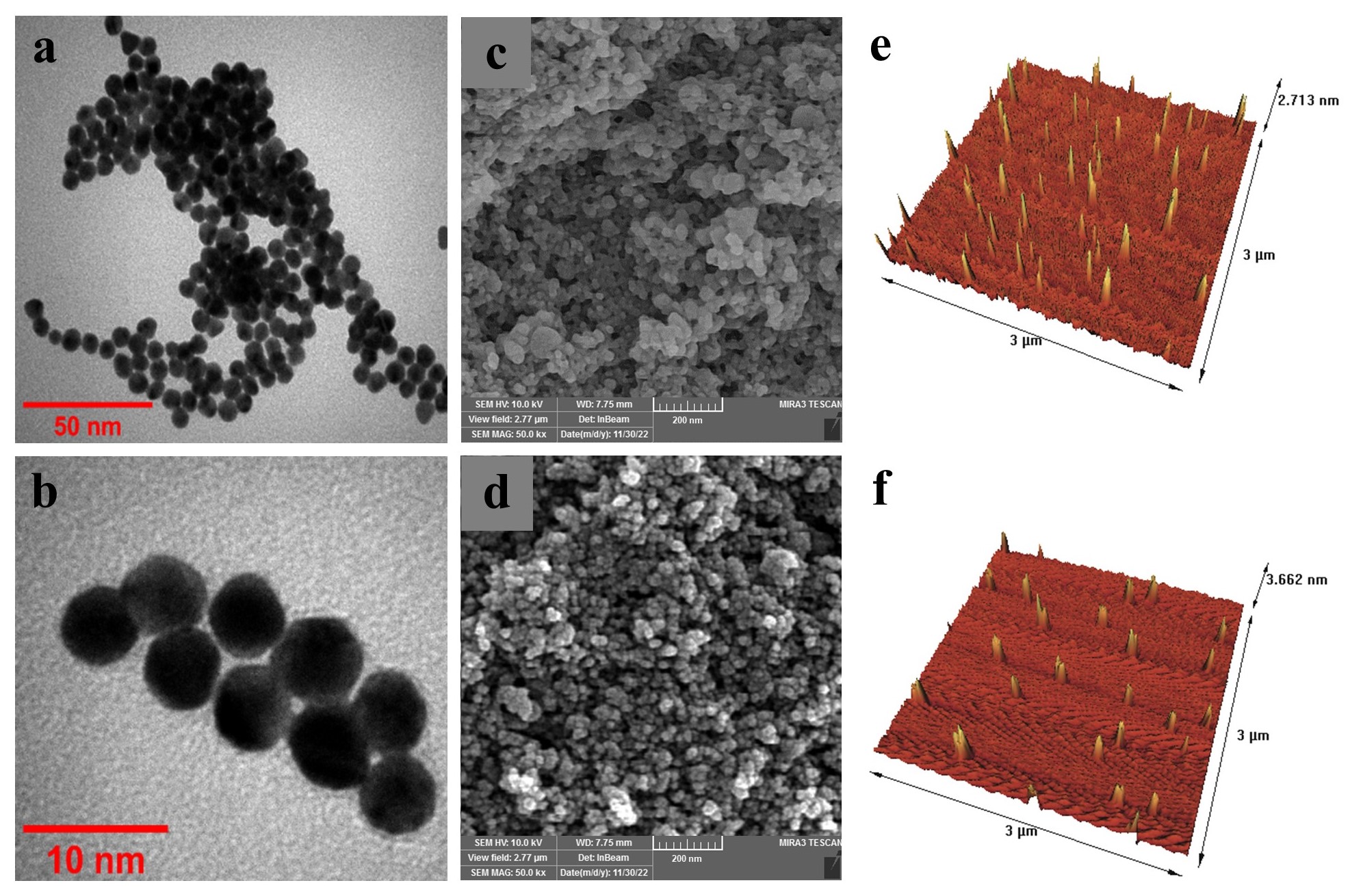


**Supplementary Fig. 1: Evaluation of structural and morphological characteristics of prepared nanoparticles.** TEM micrographs of prepared Au NPs **(a, b)**. Evaluation of morphological characteristics of QD@MSN **(c, e)** and QD@MSN-EPI-Au-PEG **(d, f)** using AFM and FE-SEM. Abbreviations: *QD*, quantum dot; *MSN*, mesoporous silica nanoparticle; *EPI*, epirubicin; *NP*, nanoparticle; *PEG*, polyethylene glycol; *TEM*, transmission electron microscopy; *AFM*, atomic force microscopy, *FE-SEM*, field emission scanning electron microscopy.

.


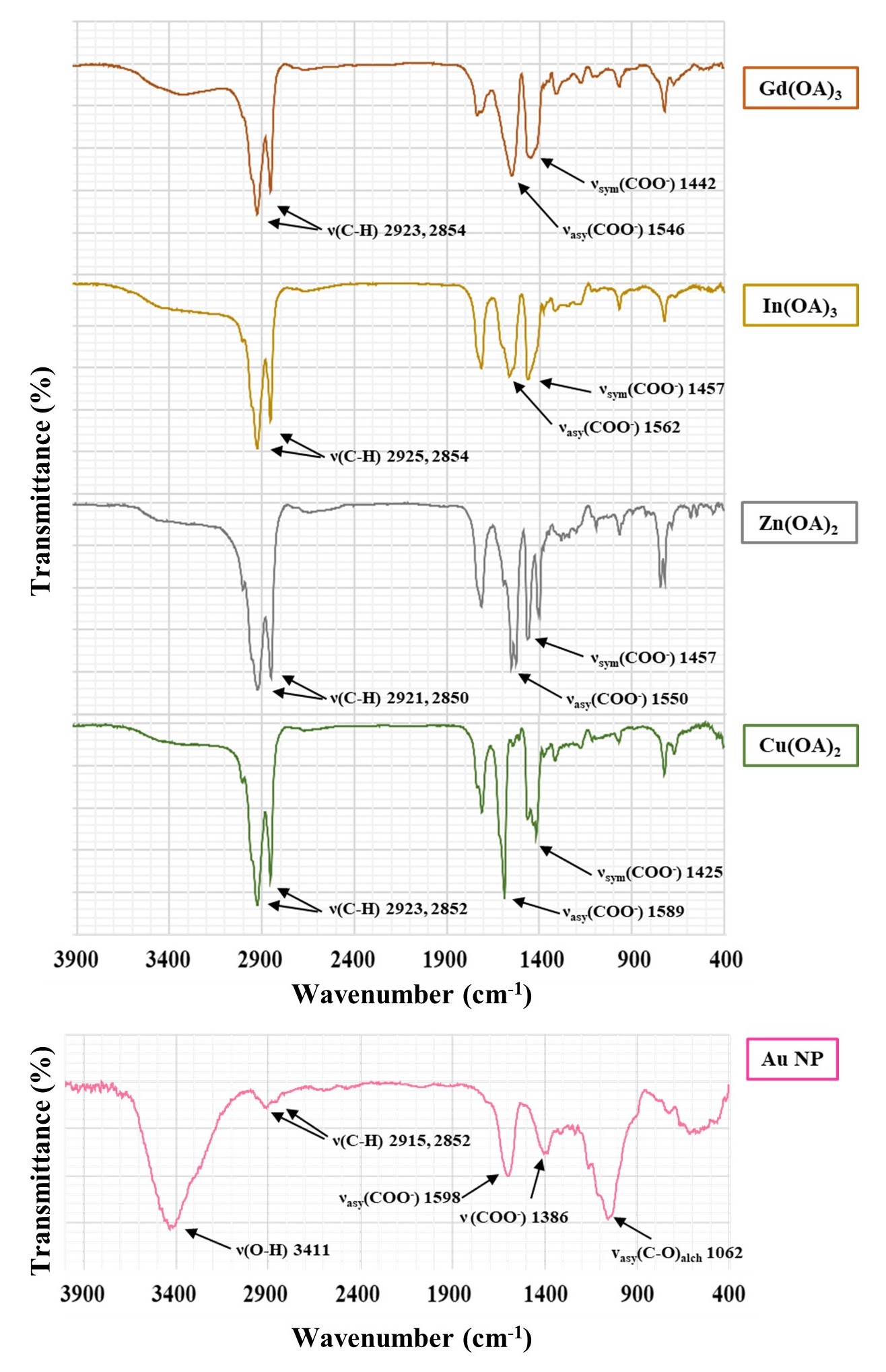


**Supplementary Fig. 2:** Fourier transform infrared spectroscopy (FT-IR) spectra analysis in each step of nanoparticles’ synthesis. Abbreviations: *OA*, oleic acid; *NP*, nanoparticle.


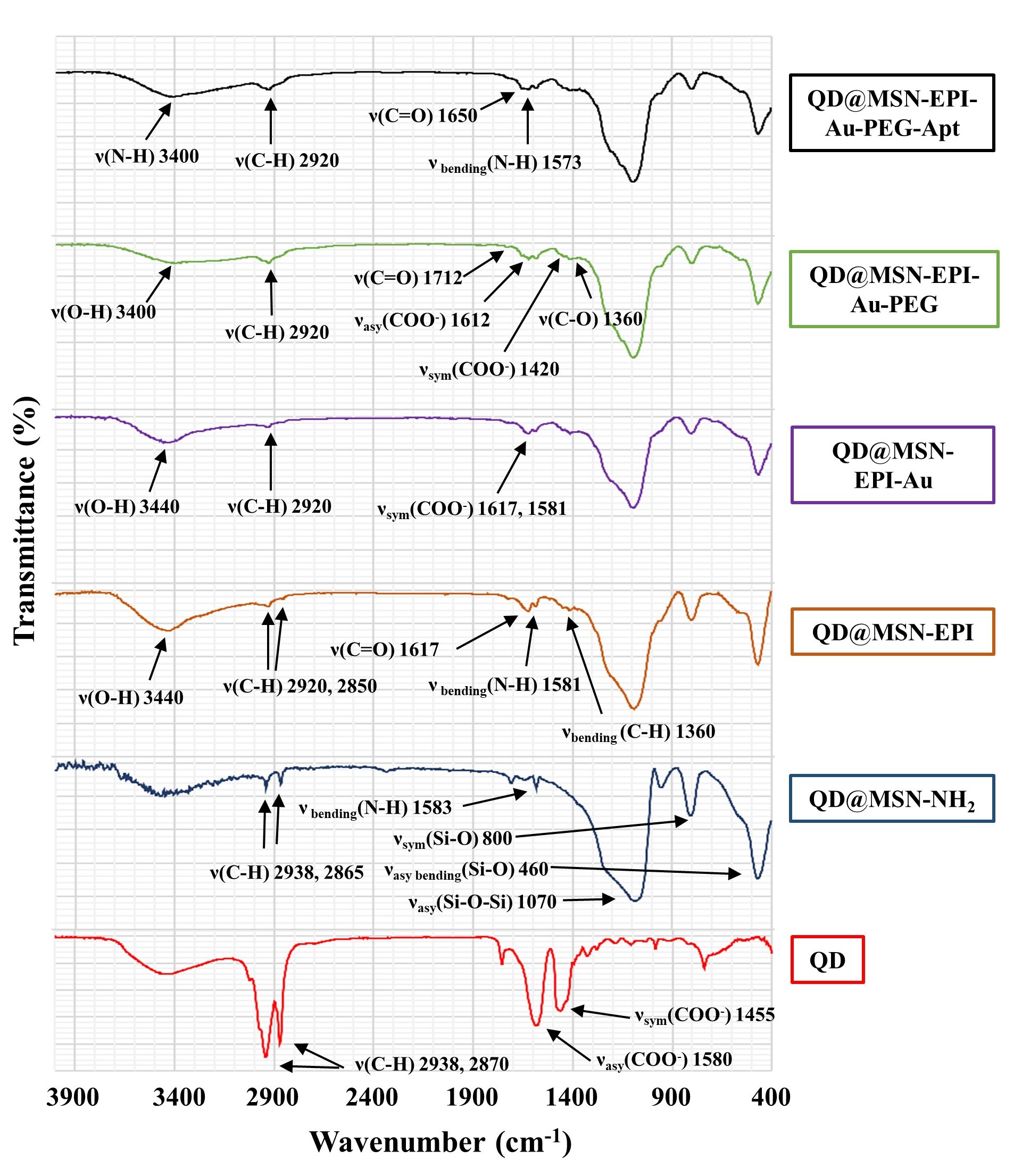


**Supplementary Fig. 3:** Fourier transform infrared spectroscopy (FT-IR) spectra analysis in each step of nanoparticles’ synthesis. Abbreviations: *QD*, quantum dot; *MSN*, mesoporous silica nanoparticle; *EPI*, epirubicin; *PEG*, polyethylene glycol; *Apt*, aptamer.


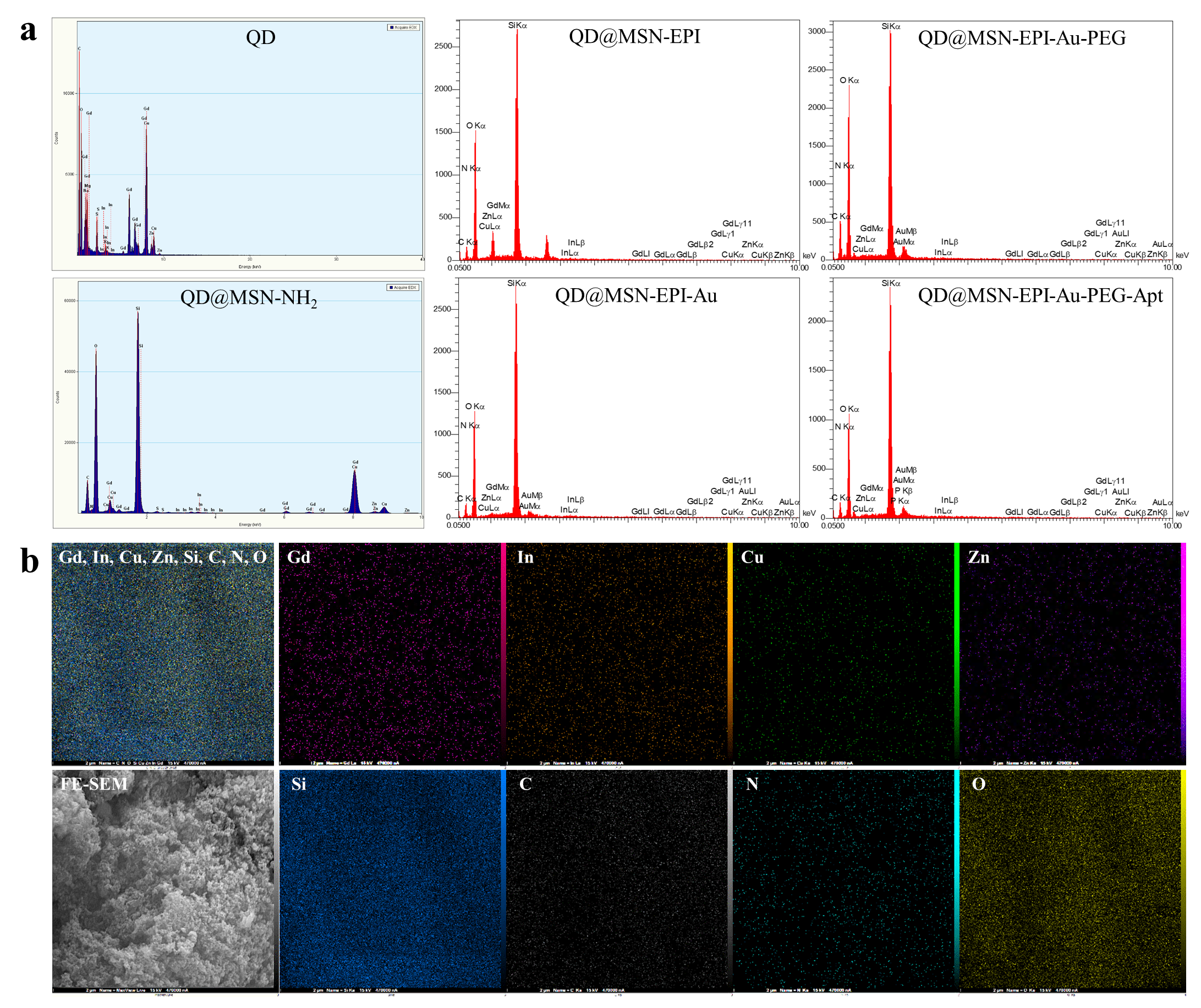


**Supplementary Fig. 4:** Energy-dispersive X-ray (EDX) spectroscopy analysis of prepared QD@MSN. EDX spectrum signals of prepared QD, QD@MSN, QD@MSN-EPI, QD@MSN-EPI-Au, QD@MSN-EPI-Au-PEG, and QD@MSN-EPI-Au-PEG-Apt **(a)**. EDX mapping of QD@MSN-NH_2_ illustrated equivalent distribution of the main elements **(b)**. Abbreviations: *QD*, quantum dot; *MSN*, mesoporous silica nanoparticle; *EPI*, epirubicin; *PEG*, polyethylene glycol; *Apt*, aptamer.


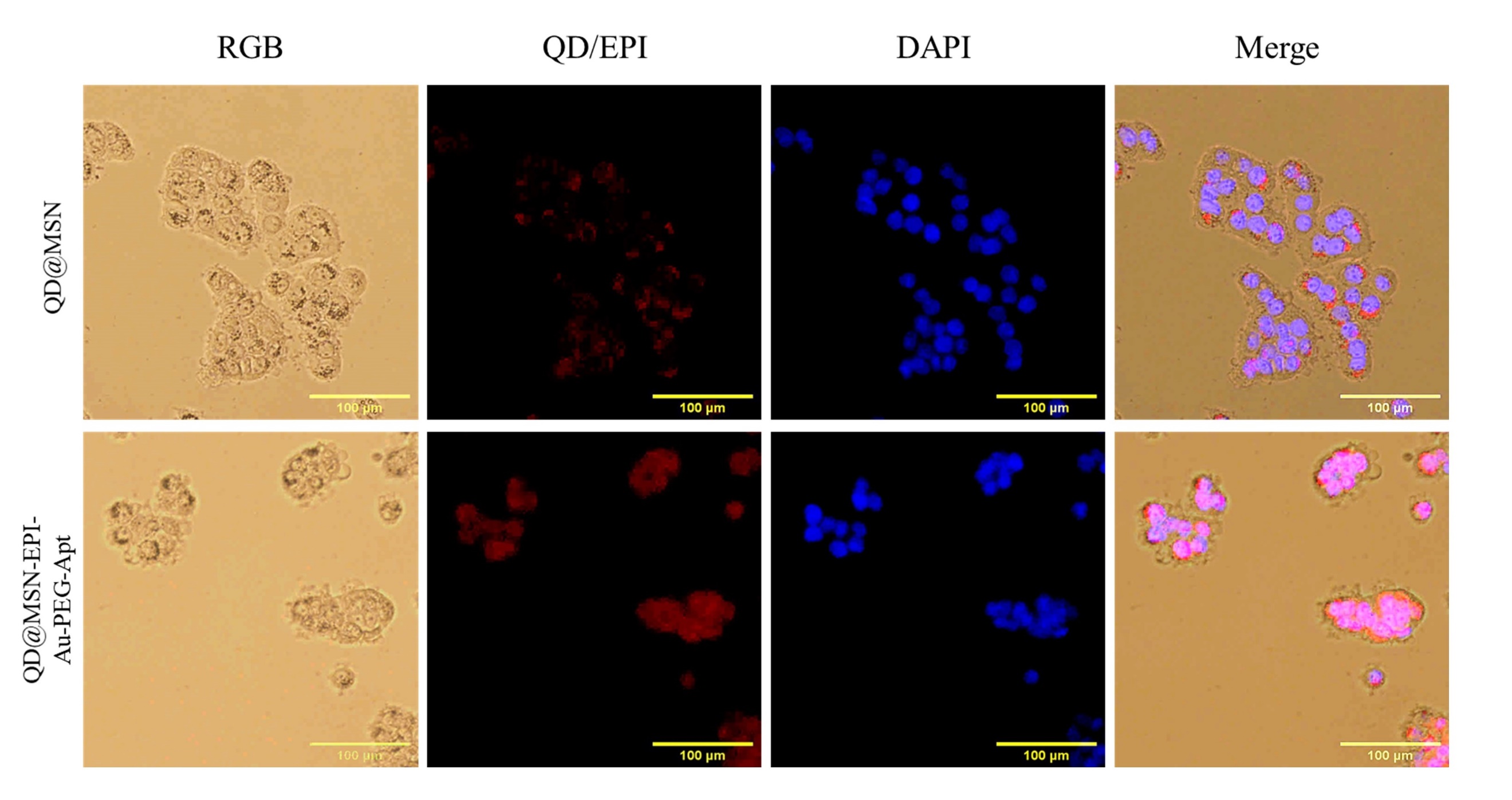


**Supplementary Fig. 5:** Qualitative uptake assessment of prepared formulations. Cellular internalization of different formulations in HT-29 cells by fluorescence microscopy. DAPI was used to stain the nuclei; scale bar: 100 µm. Abbreviations: *QD*, quantum dot; *MSN*, mesoporous silica nanoparticle; *EPI*, epirubicin; *PEG*, polyethylene glycol; *Apt*, Aptamer; *HT-29 cells*, Human colorectal adenocarcinoma cells*.*


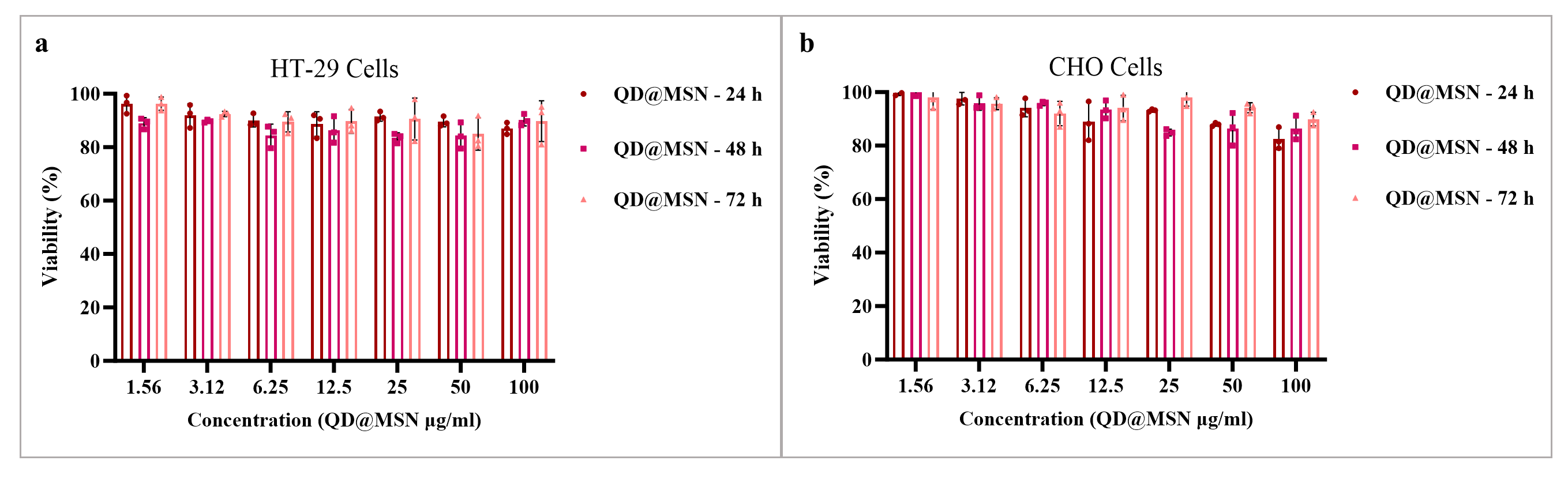


**Supplementary Fig. 6:** Cytotoxicity assessment of QD@MSN as backbone using MTT assay. Statistical comparison of different concentrations of QD@MSN against HT-29 cells after 24, 48, and 72 h **(a)** or on CHO cells following 24, 48, and 72 h **(b)**. Data are expressed as mean ± standard deviation, n = 3 biologically independent samples. Abbreviations: *QD*, quantum dot; *MSN*, mesoporous silica nanoparticle; *HT-29 cells*, Human colorectal adenocarcinoma cells*; CHO cells,* Chinese hamster ovary cells.

**Supplementary Fig. 7:** Spectrophotometry assessments for drug loading and release calculations. Calibration curve and the linear equation of the particular EPI concentration versus UV/Vis spectrophotometer absorbance.


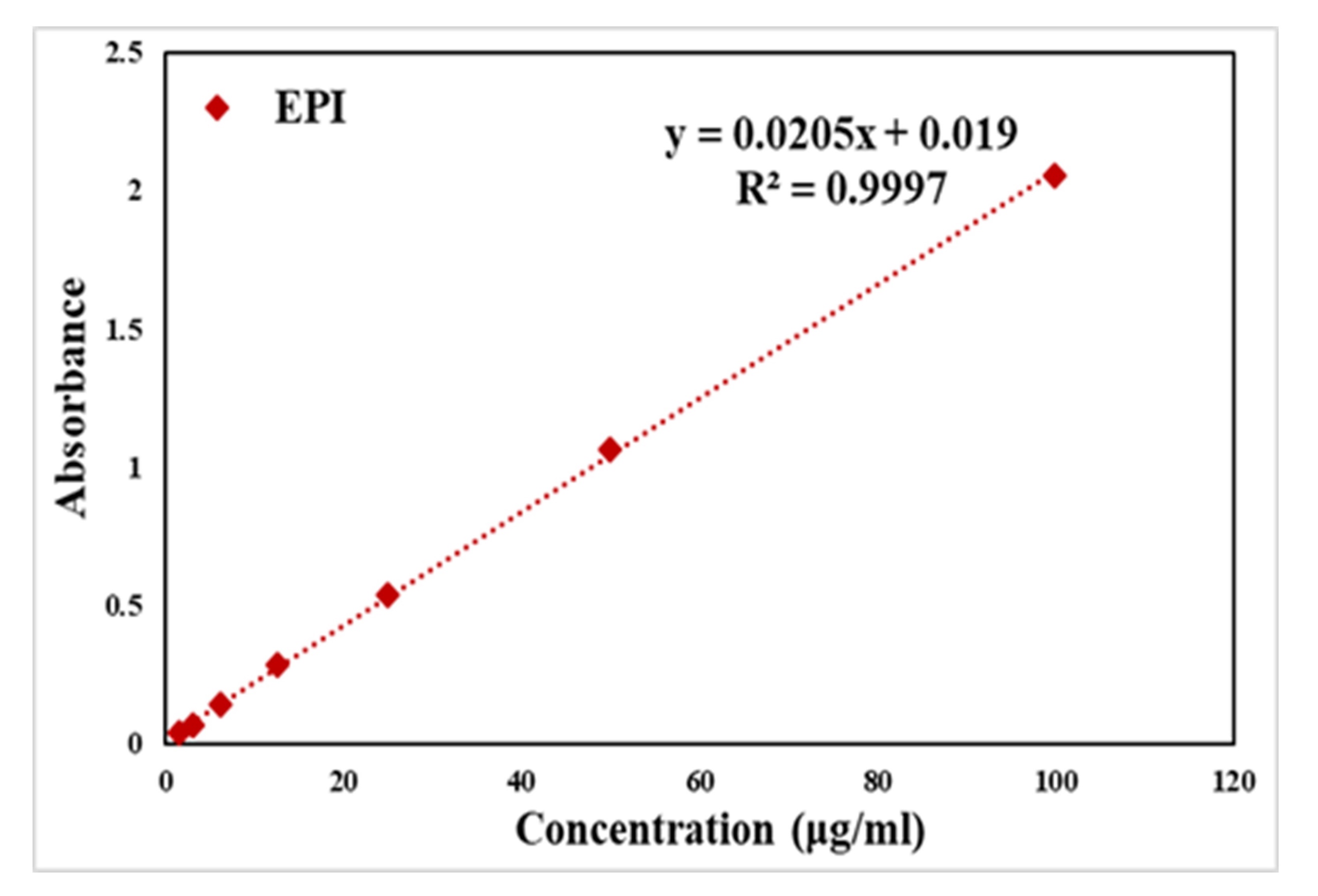

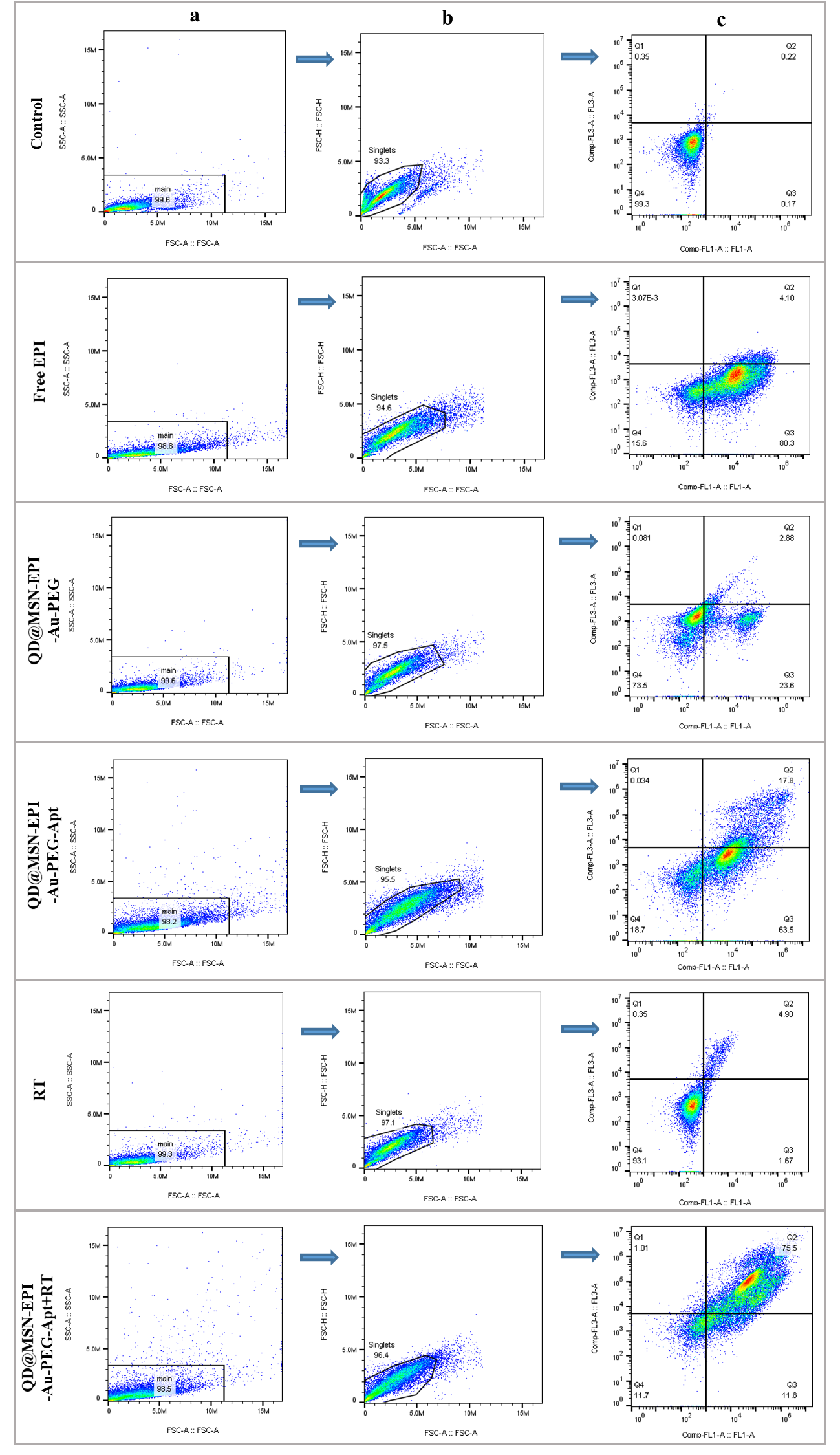


**Supplementary Fig. 8:** The gating strategy used for analyzing apoptosis with annexin-V/PI under different treatments involved several steps. Initially, HT-29 cells were gated based on forward scatter (FSC) versus side scatter (SSC) to identify the cell population **(a column)**. Subsequently, a gate was applied to FSC-Height (FSC-H) versus FSC-Area (FSC-A) to eliminate doublets and define the singlets gate **(b column)**. Finally, various subpopulations were assessed based on annexin V-FITC versus PI scatter to quantify viable, early, and late apoptotic cell populations as Q4, Q3, and Q2, respectively.

**Supplementary Tables**

Particle size was measured by dynamic light scattering (DLS). Data are expressed as mean ± standard deviation, n = 3 independent samples. Abbreviations: *QD*, quantum dot; *MSN*, mesoporous silica nanoparticle; *EPI*, epirubicin; *NP*, nanoparticle; *PEG*, polyethylene glycol; *Apt*, Aptamer.

| **Supplementary Table 1:** Mean values of size, PDI, and zeta potential of synthesized formulations in this study | | | |
| --- | --- | --- | --- |
| **Sample** | **Particle size (nm)** | **Polydispersity index (PDI)** | **Zeta potential (mV)** |
| QD@CTAB | 8.07 ± 2.95 | 0.38 ± 0.25 | +21.24 ± 1.36 |
| QD@MSN | 38.93 ± 1.32 | 0.30 ± 0.12 | -15.42 ± 1.17 |
| QD@MSN-NH_2_ | 40.72 ± 1.04 | 0.32 ± 0.06 | +27.49 ± 1.22 |
| QD@MSN-EPI | 43.02 ± 1.09 | 0.21 ± 0.11 | +24.13 ± 3.76 |
| Au NP | 5.85 ± 0.60 | 0.32 ± 0.21 | -10.22 ± 2.37 |
| QD@MSN-EPI-Au | 51.16 ± 2.94 | 0.45 ± 0.33 | +5.63 ± 1.83 |
| QD@MSN-EPI-Au-PEG | 57.54 ± 2.68 | 0.20 ± 0.02 | -13.91 ± 2.57 |
| QD@MSN-EPI-Au-PEG-Apt | 65.67 ± 3.70 | 0.28 ± 0.17 | -22.06 ± 3.86 |

| **Supplementary Table 2:** Energy-dispersive X-ray (EDX) spectroscopy analysis of prepared nanoparticles | | | | | | | | | | |  |
| --- | --- | --- | --- | --- | --- | --- | --- | --- | --- | --- | --- |
| **Samples** | **Gd** | **In** | **Cu** | **Zn** | **Si** | **Au** | **C** | **O** | **N** | **P** | |
| QD | 22.60 | 6.27 | 10.54 | 9.51 | 0 | 0 | 3.70 | 46.45 | 0.93 | 0 | |
| QD@MSN-NH_2_ | 1.79 | 0.60 | 0.45 | 0.77 | 31.46 | 0 | 10.90 | 50.01 | 4.03 | 0 | |
| QD@MSN-EPI | 0.93 | 0.43 | 0.32 | 0.47 | 21.03 | 0 | 15.77 | 52.59 | 5.16 | 0 | |
| QD@MSN-EPI-Au | 1.18 | 0.49 | 0.31 | 0.49 | 20.99 | 10.49 | 16.34 | 44.51 | 5.19 | 0 | |
| QD@MSN-EPI-Au-PEG | 0.55 | 0.41 | 0.29 | 0.23 | 14.24 | 7.00 | 22.84 | 47.55 | 6.89 | 0 | |
| QD@MSN-EPI-Au-PEG-Apt | 1.01 | 0.57 | 0.48 | 0.50 | 20.68 | 12.51 | 16.10 | 43.44 | 4.53 | 0.19 | |

The data are represented as weight percentage (W%). Abbreviations: *QD*, quantum dot; *MSN*, mesoporous silica nanoparticle; *EPI*, epirubicin; *PEG*, polyethylene glycol; *Apt*, Aptamer.

| **Supplementary Table 3:** Mean values of size and PDI of QD@MSN-EPI-Au-PEG-Apt after incubation for 4, 24, and 48 h | | | | | | |
| --- | --- | --- | --- | --- | --- | --- |
| **Media** | **PBS** | | | **PBS containing 30% FBS** | | |
|  | **4 h** | **24 h** | **48 h** | **4 h** | **24 h** | **48 h** |
| **Particle size (nm)** | 66.54 ± 1.10 | 68.60 ± 4.21 | 72.24 ± 3.22 | 69.18 ± 2.07 | 72.27 ± 3.75 | 86.31 ± 3.28 |
| **Polydispersity index (PDI)** | 0.19 ± 0.01 | 0.28 ± 0.15 | 0.38 ± 0.20 | 0.24 ± 0.17 | 0.33 ± 0.30 | 0.33 ± 0.29 |

Particle size was measured by dynamic light scattering (DLS) in PBS and FBS (30%, v/v). Data are expressed as mean ± standard deviation, n = 3 independent samples. Abbreviations: *QD*, quantum dot; *MSN*, mesoporous silica nanoparticle; *EPI*, epirubicin; *NP*, nanoparticle; *PEG*, polyethylene glycol; *Apt*, Aptamer.

Data are expressed as mean ± standard deviation, n = 3 biologically independent samples. Abbreviations: *QD*, quantum dot; *MSN*, mesoporous silica nanoparticle; *EPI*, epirubicin; *PEG*, polyethylene glycol; *Apt*, Aptamer.

| **Supplementary Table 4:** IC_50_ values of different nanocarriers on HT-29 and CHO cells during 24, 48, and 72 h | | | | | | |
| --- | --- | --- | --- | --- | --- | --- |
| **Treatments** | **IC_50_ (µg/ml) ± SD (HT-29 cells)** | | | **IC_50_ (µg/ml) ± SD (CHO cells)** | | |
|  | **24 h** | **48 h** | **72 h** | **24 h** | **48 h** | **72 h** |
| Free EPI | 5.31 ± 1.30 | 2.31 ± 1.07 | 1.11 ± 0.92 | 6.07 ± 0.95 | 2.03 ± 0.73 | 0.94 ± 0.67 |
| QD@MSN-EPI-Au-PEG | 17.12 ± 1.67 | 5.20 ± 1.45 | 1.76 ± 0.91 | 28.03 ± 1.76 | 21.51 ± 1.06 | 6.54 ± 1.28 |
| QD@MSN-EPI-Au-PEG-Apt | 3.26 ± 1.36 | 1.84 ± 0.84 | 0.95 ± 0.75 | 42.04 ± 0.97 | 31.18 ± 0.87 | 10.62 ± 1.14 |

**Supplementary Discussion**

In this study, the targeted nanocarrier as QD@MSN-EPI-Au-PEG-Apt was first designed and synthesized successfully and the fundamental steps of synthesis process were fully characterized. The absorbance and fluorescence emission of the bare QDs as well as mesoporous silica coated QDs using PL and UV/Vis spectroscopy indicated excellent optical properties. The influence of magnetic field on prepared QD and QD@MSN were evaluated by VSM and MRI as well as X-ray attenuation associated to QD and Au NP in CT scan and the results brightened second and third imaging modality capabilities. DLS and ELS analysis demonstrated size and zeta potential alterations in each step of preparation, and the results of HR-TEM, FE-SEM, and AFM confirmed the spherical shape and monodispersed morphology of prepared QDs, QD@MSNs and Au NPs. Further physicochemical characterizations such as XRD, TGA, BET, BJH, FT-IR and EDX clearly confirmed surface modifications and structure, pore volume, functional groups and elemental compositions of nanoparticles, respectively.

The optical analysis of obtained GZCIS/ZnS QDs suggested the high PL quantum yield (QY) around 40%, representing sufficient FL, as demonstrated by Guo *et al*. the controlled PL qualities can be precisely customized by adjusting the Zn/Cu ratio with good reproducibility since there is little influence of altering reaction time and Gd/Cu ratio on the PL emission^1^. Similarly, other studies are in agreement with synthesis of Zn–Cu–In–S (ZCIS) and ZCIS/ZnS quaternary QDs, as a derivative formulation of CuInS_2_ QDs, in terms of bright fluorescence and color tunability by adjusting size and Zn/Cu feeding ratio^2,3^. Meanwhile, the dramatical enhancement of the GZCIS QDs FL (PL QY from 10 to 40%) became possible by *in situ* growth of a ZnS shell in an effort to passivate inorganic surfaces^1,4^. Other studies declared the same PL efficiency improvement by hybridizing ZnS materials with CuInS_2_ nanoparticles as an inorganic shell for surface passivation which led to a wide direct band gap for confining electrons and holes in the core^2,5^. Moreover, the use of paramagnetic ion (Mn^2+^ and Gd^3+^) doped QDs as dual-modal imaging probes has become increasingly popular in recent years^1^. For instance, Yang *et al.* fabricated an optimized CuInS/ZnS bimodal quantum dot (BQD) with Gd doping^6^ and Wang *et al*. exhibited various size of core/shell CdSe/Zn_1–x_ Mn_x_S nanoparticles and Mn^2+^ content^7^. Although, Guo *et al*. observed a slight decrease in PL QYs after the introduction of Gd species^1^, the CIS-based QDs emit more PL in the presence of more intrinsic defects like copper vacancies and In or Gd cations on Cu antisites due to a donor–acceptor pair (DAP) recombination mechanism^8^.

While synthesis of hydrophobic QDs opposes their direct bio-applications, QD production in organic solvents followed by a phase transition to the aqueous phase provides some advantages such as superior optical properties^9^. Since we used silica coating for QD phase transfer from organic to aqueous while retaining most of its PL emission properties, surface silanization was performed in accordance with several studies to increase QDs solubility in aqueous media due to anomalous behavior of silica in water and strong stability at near neutral pH even under high salt concentrations^10^. Interestingly, red shifts in PL emission from silica coated QDs observed in our results, was in agreement with previous reports of synthesized QD@MSN by the reverse microemulsion method which was ascribed to ligand substitution and surface charge alteration^5,11,12^. The used microemulsion assisted sol-gel method not only embeds QDs in silica shell, but also provides a mesoporous structure with high surface area to combine FL-MR agents with chemotherapeutic drugs as a theranostic platform. To this aim, the comparison of optical, magnetic, physical, and structural properties of mesoporous silica coated QDs with bare QDs were performed. Notably, the size and zeta potential of QD@CTAB were turned from 8.43 nm and +21.14 mV to 39.21 nm and -15.62 mV in their QD@MSN form, and then to 41.06 nm and +27.52 mV in QD@MSN-NH_2_ form, respectively. This confirms the successful completion of the silica coating and amination procedures. The structural properties of QD and QD@MSN illustrated similarities with the TEM results reported by Lin *et al*. and Kim *et al*. who fabricated multicore QD@MSN^5,13,14^. Although the peaks related to QDs in XRD pattern were broadened due to small dimensions, the synthesized QDs are found to be in mixed of rhombohedral, cubic, and hexagonal blend phase according to reference database used PDF-4+2018RDB^15^. The TGA results of three important steps of synthesis evaluated and confirmed the weight loss patterns, as represented by previous studies for QDs^9,16,17^, silica^18^, and PEG^19^.

After encapsulating the EPI in open pores of QD@MSN with EE% and LC% of 70% ± 1.56 and 25% ± 1.43, respectively, Au NPs were incorporated via electrostatic interactions between citrate ions of Au NPs and amine groups of MSNs^20^. The successful pore capping of the MSNs was demonstrated by BET surface area and BJH pore volume reduction of about 272.59 m^2^/g and 3.07 cm^3^/g which was accordant with similar gold capped MSNs prepared by Iranpour *et al*. and Al-mosawi *et al*.^21,22^. The release profile of gold-capped nanocarriers was investigated at pH 5.4, 6.4, and 7.4 representing endosomes, TME, and physiological body fluids. Based on the obtained results, the most cumulative release (70.03%) after 6 days occurred at pH=5.4 which was significantly higher and faster than that at pH 6.4 and 7.4. The intelligent drug release behavior of DDSs with gold gatekeepers in acidic pH occurs because of amine groups protonation on the surface of MSN and subsequently dissociation of gold gatekeepers similar to previous reports^20–22^. In this regard, Ming *et al*. used gold nanorods as gate keeping agents of MSN in order to control DOX release at acidic and physiological pH^23^.

The successful coating of nanocarriers with PEG polymer by thiol–Au linkage between PEG and gold gatekeeper, was confirmed by enhancement of particle size (~8 nm) and weight loss (~38%) as well as presence of functional groups (-COOH) and elemental composition (O and C) by the DLS, TGA, FT-IR, and EDX results, respectively. Notably, nanoparticles coated with PEG, showed improved solubility and dispersibility as well as circulatory half-life and blood biocompatibility enhancement^24^. Furthermore, hemolysis assay illustrated a negligible impact on RBC lysis for PEGylated nanoparticles in contrast to uncoated NPs (Fig. 4). These results are in line with several studies, which were conducted to investigate the influence of PEG coating on physicochemical properties of MSNs^25^. In this regard, Suk *et al*. reviewed the studies and mechanisms that considered aggregation, opsonization, phagocytosis, and prolonging systemic circulation time of nanoparticles after coating with PEG as well as methods for effective synthesis and characterization of PEGylated nanoparticles^26^. Moreover, Desai *et al*. and He *et al*. illustrated that PEGylation of MSNs could improve the blood half-life circulation via lower entrapment in organs and better penetration into epithelial cells^27,28^. Based on our results, PEGylation of nanocarriers (QD@MSN-EPI-Au-PEG) led to repression of hemolytic properties of MSNs to <5%. Studies have considered hemolysis less than 10% as acceptable threshold of biocompatibility for *in vivo* experiments^29,30^.

The last step of synthesis was conjugating EpCAM aptamer to PEGylated nanocarriers via an EDC/NHS reaction involving the amine group of EpCAM aptamer and the carboxylic acid group of heterofunctional PEG, which was confirmed by zeta potential and particle size alterations, amide band in FT-IR spectrum and presence of phosphor signal in EDX. Notably, the comparison between targeted and non-targeted nanoparticles in agarose gel electrophoresis illuminated successful decoration of nanocarriers with EpCAM aptamer. Since the prepared nanocarriers remained in the well due to their heavy weight, the bright band related to QD@MSN-EPI-Au-PEG-Apt demonstrated the presence of aptamer on their surface. In order to investigate targeting performance of prepared nanocarriers *in vitro*, cellular internalization of nanocarriers in comparison with Free EPI was evaluated on both HT-29 EpCAM-positive and CHO as EpCAM-negative cells. The obtained results using fluorescent microscopy and flow cytometry illuminated the higher uptake of QD@MSN-EPI-Au-PEG-Apt by HT-29 compared to CHO cells whereas no significant difference was observed for the uptake of QD@MSN-EPI-Au-PEG and QD@MSN in the two cell lines (Fig. 5). The overexpression of EpCAM on the surface of CRC cells has been demonstrated in numerous studies^31,32^, which suggests that nanoparticles equipped with specific ligands can be used to actively deliver their cargos to cancer cells via receptor-mediated endocytosis. Our findings were in agreement with several studies using EpCAM aptamer as targeting agent to deliver nanocarriers selectively to cancerous cells^33–36^. For instance, Li *et al*. synthesized PDA coated MSNs conjugated with PEG and EpCAM aptamer to increase binding ability to the CRC SW480 cell line^37^.

In order to investigate the therapeutic efficiency of targeted drug loaded-nanocarriers in combination with radiotherapy, the cytotoxicity of prepared formulations was compared both *in vitro* and *in vivo*. MTT results illustrated the anti-cancer efficacy of Free EPI, QD@MSN-EPI-Au-PEG, and QD@MSN-EPI-Au-PEG-Apt in terms of chemotherapy treatment before introducing radiation therapy, however no toxicity related to other parts of nanoparticle was observed. The results showed a) a significant anti-cancer activity enhancement of targeted nanocarriers compared to non-targeted ones against HT-29 cells during 24, 48, and 72 h, b) a decrease in viability of HT-29 cells with increasing time and dosage, c) QD@MSN-EPI-Au-PEG-Apt showed excellent anti-cancer activity against HT-29 cells while it did not cause any noticeable cytotoxicity against CHO cells, and d) similar cytotoxic effects of Free EPI on both EpCAM-positive and negative cells. According to these findings, EpCAM aptamer as the targeting moiety plays a critical role in recognizing receptors on cancer cells and selectively delivery of EPI, while Free EPI could cause severe side effects due to its nonspecific delivery. In this regard, other studies have acknowledged that the therapeutic response of chemotherapy could be improved by specific penetration of NPs within the tumor cells^38,39^. For instance, Jalalian *et al*. conducted EPI loaded NPs with 5TR1 aptamer as the target agent to enhance the toxicity in MCF-7 (human breast carcinoma cell) and C26 (murine colon carcinoma cell) cancerous cells^40^. On the other hand, the combinational therapeutic approach was first investigated via colony formation of both HT-29 and CHO cells after introducing radiotherapy. The obtained results from colony formation assay (CFA) exhibited a remarkable reduction in HT-29 survival fraction (SF) at 3 and 6 Gy radiation doses, only when the cells were pre-treated with QD@MSN-EPI-Au-PEG-Apt. Apart from dependence of SF to both radiation dose and NPs concentration in HT-29 cells, the slight reduction of SF in CHO cells confirmed that different combinatorial effects in the two cell lines is due to specific internalization of targeted NPs into HT-29 cells. The complete structure of suggested nanoparticle (QD@MSN-EPI-Au-PEG-Apt) contains Au and Gd which both belong to high atomic number (Z) elements with interesting properties in X-ray radiosensitization and CT scan^41–43^. As reviewed by Chen *et al*., numerous studies evaluated different aspects of Au NPs in terms of sensitizing the radiotherapy effectiveness via physical, chemical, and biological mechanisms, so that the radiation dose can be decreased to minimize the side effects of radiotherapy on normal tissues^44^. Further analysis of this phenomenon is based on a Monte Carlo simulation, which indicates that inhomogeneity in the distribution of gold atoms due to size might influence secondary ionization^45^. In this regard, the fundamental and efficiency of Au NPs for RT sensitization enhancement in various radiation doses (2-8 Gy) were confirmed by researchers *in vitro* and *in vivo*, however neither one explained why the dose enhancement factor was calculated at that dose level^46^. In line with our study, Coulter *et al*. showed that employing a single 3 Gy dose of 6 MV X-ray in combination with gold nanoparticle treatment sensitized cell line models as demonstrated by colony-forming assay^47^. Notably, using 6 MV X-ray in the presence of Au NPs led to significant increase in inhibition of cancer cells^48,49^ as Xu *et al*. demonstrated that exposure of radioresistant melanoma cells to Au nanorods and MV X-rays led to deduction of integrin expression and rendered the cells susceptible to radiation-induced apoptosis^50^. Although the cell death mechanism results clearly illuminated that more population of apoptotic HT-29 cells were associated to targeted nanocarriers compared to non-targeted treatment group, the increased late apoptosis rate induced by QD@MSN-EPI-Au-PEG-Apt+RT to about 60% confirmed the effectiveness of combinatorial therapeutic approaches. In this regards, Zhang *et al*. treated LS180 (colorectal cancer cell line) with Au NPs which were functionalized with R8 and PEG in combination with megavoltage radiotherapy. In their study, using R8 as a transmembrane vector resulted in efficient internalization and cellular uptake of Au NPs, and irradiation with 6 MV X-ray led to enhancement of apoptosis and ROS levels as well as surviving fraction deduction^51^.

In order to investigate the anti-tumor efficacy and possible side effects of combinational therapeutic approach, the second part of the biological experiments was conducted on immunocompromised C57BL/6 mice bearing human HT-29 tumors. As shown at cellular level, *in vivo* results exhibited a significant suppression of tumor growth by targeted nanocarriers compared to non-targeted and control groups. Notably, in combinational treatment group (QD@MSN-EPI-Au-PEG-Apt+RT) a remarkable reduction of tumor size occurred which approximately led to tumor elimination via high level of apoptosis (Fig. 7). Furthermore, the histological evaluation in addition to body and liver weight results not only confirmed the systemic toxicity and severe side effects of Free EPI, but also illustrated the negligible side effects of EPI when it was encapsulated in nanocarriers (Fig. 8). Several studies have demonstrated the efficacy of targeted nanocarriers in tumor growth retardation with no noticeable side effects as reviewed by Karimi *et al*.^52^. On the other hand, the effect of radiotherapy in combination with Au NPs which increase the anti-tumor efficiency via radiosentisization to ionizing radiation, has been studied previously^53,54^. For instance, Liu *et al*. evaluated radiosensitization effects of bovine serum albumin-templated gold nanoparticles (BSA-GNPs) in different sizes via intravenous injection with a dose of 4 mg Au/kg and 5 Gy X-ray irradiation, and the results indicated an enhancement factor of about 2^55^.

Apart from the therapeutic aspects of prepared nanocarriers, their potential application in imaging modalities including FL, MR, and CT imaging was also investigated. The fluorescence *ex vivo* imaging of tumor and main organs was performed to consider fluorescence feasibility of QDs combined with EPI in the body of C57BL/6 mice bearing human HT-29 tumors. The quantitative and qualitative results of FL revealed the accumulation of targeted nanocarriers in tumors while the Free EPI was distributed in all organs. These findings confirm that both passive and active targeting through EPR effect and EpCAM aptamer mediated drug delivery, respectively, are active. These observations were in agreement with MRI and CT scan results. Since the quantitative imaging characteristics of nanoparticles containing Gd and Au were confirmed (Fig. 2), the *in vivo* imaging efficacy due to positive contrasting ability of QDs and Au NPs illuminated biodistribution of targeted and non-targeted nanocarriers. At this stage, QDs containing gadolinium as *T_1_* MR contrast agent were recently developed for dual FL and MR imaging by several studies^6,56,57^. For instance, Zavvar *et al*. utilized both fluorescence and magnetic properties of QDs (Gd-Zn-Cu-In-S/ZnS) equipped with DOX as therapeutic and AS1411 aptamer as active targeting moiety for theranostic purposes^15^. Furthermore, gold nanoparticles with strong X-ray attenuation coefficient due to the high atomic number of Au element could represent a high contrast between healthy tissues and tumors when the gold nanoparticles accumulate in tumors on CT images. Numerus studies demonstrated Au NPs application in CT imaging as a preferred contrast agent due to their nontoxicity and facile synthesis as well as their capability for surface modification in drug delivery^48,49,58,59^. In this regard, Dou *et al*. reported that Au NPs in the size range of 3-50 nm provided a significant size-dependent enhancement in CT images as well as radiotherapy^45^. It should be noted that the biodistribution of nanocarriers illuminated by FL, MR, and CT imaging revealed less intensity both in organs and tumor at the second time point which was 12 h longer than the first time. Similarly, Liu et al. showed the highest accumulation of HB@VHMBi-Gd in tumors at 12 h post-injection by the *Fe*-weighted MR and photoacoustic imaging which thereafter, the MR signals began to decrease^60^. Although according to this finding the optimized time for tumor accumulation is lower than 24 h post injection, we irradiated the animals 24 h post-injection with 6 MV X-ray exposure in a combinatorial approach to avoid the possible side effects mediated via radiosensitization effects of nanoparticles in healthy tissues.

Previous studies suggest that inorganic nanoparticles larger than 6 nm are typically eliminated through hepatobiliary and feces excretion, while smaller nanoparticles below 5.5 nm size are efficiently excreted through the urinary system^61^. Amorphous silica, commonly used to coat nanoparticles, improves biocompatibility and degrade and clear at rates dependent on size, shape, and surface functionalization^62^. Au NPs smaller than 5-10 nm can be eliminated through renal filtration, while larger non-biodegradable nanoparticles or biodegradable nanocarriers may undergo breakdown or metabolism, either returning to circulation or being retained in liver cells^63^. Therefore, the hybridized nanoplatform could potentially undergo multiple mechanisms for degradation and elimination from the body.

In summary, we first designed and synthesized a multimodal nanocarrier with interesting characteristics by combining several strategies. Afterward, the prepared nanocarriers were evaluated in terms of physicochemical properties and biological performance *in vitro* and *in vivo* in order to assess their anti-cancer properties comprehensively. Overall, GZCIS/ZnS quaternary QDs as semiconductor nanocrystals with excellent fluorescence and magnetic properties were used as the core of mesoporous silica shell to use their benefits in traceability and high drug loading capacity for simultaneous cancer therapy and imaging as a theranostic DDS. Au NPs were hybridized as pH-sensitive gatekeepers, radiosensitizer, and CT contrast agents and incorporated with PEG to increase biocompatibility and blood circulation. Moreover, the intelligent structure armed with EpCAM aptamer optimized the specific delivery to HT-29 cancer cells via active targeting mechanism. The *in vitro* and *in vivo* results illustrated excellent anti-cancer as well as imaging properties of QD@MSN-EPI-Au-PEG-Apt as a theranostic formulation.

**Supplementary References**

1. Guo, W. *et al.* Color-tunable Gd-Zn-Cu-In-S/ZnS quantum dots for dual modality magnetic resonance and fluorescence imaging. *Nano Res.* **7**, 1581–1591 (2014).

2. Zhang, J., Xie, R. & Yang, W. A simple route for highly luminescent quaternary Cu-Zn-In-S nanocrystal emitters. *Chem. Mater.* **23**, 3357–3361 (2011).

3. Zhang, W. & Zhong, X. Facile synthesis of ZnS-CuInS_2_-alloyed nanocrystals for a color-tunable fluorchrome and photocatalyst. *Inorg. Chem.* **50**, 4065–4072 (2011).

4. Guo, W. *et al.* Synthesis of Zn-Cu-In-S/ZnS core/shell quantum dots with inhibited blue-shift photoluminescence and applications for tumor targeted bioimaging. *Theranostics* **3**, 99–108 (2013).

5. Lin, B. *et al.* Multifunctional gadolinium-labeled silica-coated core/shell quantum dots for magnetic resonance and fluorescence imaging of cancer cells. *RSC Adv.* **4**, 20641–20648 (2014).

6. Yang, W. *et al.* Facile synthesis of Gd-Cu-In-S/ZnS bimodal quantum dots with optimized properties for tumor targeted fluorescence/MR *in vivo* imaging. *ACS Appl. Mater. Interfaces* **7**, 18759–18768 (2015).

7. Wang, S., Jarrett, B. R., Kauzlarich, S. M. & Louie, A. Y. Core/shell quantum dots with high relaxivity and photoluminescence for multimodality imaging. *J. Am. Chem. Soc.* **129**, 3848–3856 (2007).

8. Chen, B. *et al.* Highly emissive and color-tunable CuInS2-based colloidal semiconductor nanocrystals: Off-stoichiometry effects and improved electroluminescence performance. *Adv. Funct. Mater.* **22**, 2081–2088 (2012).

9. Heyne, B. *et al.* Mixed mercaptocarboxylic acid shells provide stable dispersions of InPZnS/ZnSe/ZnS multishell quantum dots in aqueous media. *Nanomaterials* **10**, 1–24 (2020).

10. Karakoti, A. S., Shukla, R., Shanker, R. & Singh, S. Surface functionalization of quantum dots for biological applications. *Adv. Colloid Interface Sci.* **215**, 28–45 (2015).

11. Shen, J. *et al.* Multifunctional gadolinium-labeled silica-coated Fe_3_O_4_ and CuInS_2_ nanoparticles as a platform for *in vivo* tri-modality magnetic resonance and fluorescence imaging. *J. Mater. Chem. B* **3**, 2873–2882 (2015).

12. Akbarzadeh, M. *et al.* Hybrid silica-coated Gd-Zn-Cu-In-S/ZnS bimodal quantum dots as an epithelial cell adhesion molecule targeted drug delivery and imaging system. *Int. J. Pharm.* **570**, 118645 (2019).

13. Pham, X. H. *et al.* Synthesis and application of silica-coated quantum dots in biomedicine. *Int. J. Mol. Sci.* **22**, (2021).

14. Kim, J. *et al.* Magnetic fluorescent delivery vehicle using uniform mesoporous silica spheres embedded with monodisperse magnetic and semiconductor nanocrystals. *J. Am. Chem. Soc.* **128**, 688–689 (2006).

15. Zavvar, T. S. *et al.* Synthesis of multimodal polymersomes for targeted drug delivery and MR/fluorescence imaging in metastatic breast cancer model. *Int. J. Pharm.* **578**, 119091 (2020).

16. Wada, C., Iso, Y., Isobe, T. & Sasaki, H. Preparation and photoluminescence properties of yellow-emitting CuInS_2_/ZnS quantum dots embedded in TMAS-derived silica. *RSC Adv.* **7**, 7936–7943 (2017).

17. Wang, Z. *et al.* Facile synthesis of Cu-In-S/ZnS core/shell quantum dots in 1-dodecanethiol for efficient light-emitting diodes with an external quantum efficiency of 7.8%. *Chem. Mater.* **30**, 8939–8947 (2018).

18. Premaratne, W., Priyadarshana, W., Gunawardena, S. H. P. & De Alwis, A. A. P. Synthesis of nanosilica from paddy husk ash and their surface functionalization. *Univ. Kelaniya* (2013).

19. Chieng, B. W., Ibrahim, N. A., Yunus, W. M. Z. W. & Hussein, M. Z. Poly(lactic acid)/poly(ethylene glycol) polymer nanocomposites: Effects of graphene nanoplatelets. *Polymers (Basel).* **6**, 93–104 (2014).

20. Babaei, M. *et al.* Synthesis of theranostic epithelial cell adhesion molecule targeted mesoporous silica nanoparticle with gold gatekeeper for hepatocellular carcinoma. *Nanomedicine* **12**, 1261–1279 (2017).

21. Iranpour, S., Bahrami, A. R., Nekooei, S., Saljooghi, A. S. & Matin, M. M. Improving anti‑cancer drug delivery performance of magnetic mesoporous silica nanocarriers for more efficient colorectal cancer therapy. *J. Nanobiotechnology* 1–23 (2021).

22. Kamil Mohammad Al-Mosawi, A., Bahrami, A. R., Nekooei, S., Saljooghi, A. S. & Matin, M. M. Using magnetic mesoporous silica nanoparticles armed with EpCAM aptamer as an efficient platform for specific delivery of 5-fluorouracil to colorectal cancer cells. *Front. Bioeng. Biotechnol.* **10**, 1095837 (2023).

23. Ma, M. *et al.* Au capped magnetic core/mesoporous silica shell nanoparticles for combined photothermo-/chemo-therapy and multimodal imaging. *Biomaterials* **33**, 989–998 (2012).

24. Knop, K., Hoogenboom, R., Fischer, D. & Schubert, U. S. Poly(ethylene glycol) in drug delivery: pros and cons as well as potential alternatives. *Angew. Chem. Int. Ed. Engl.* **49**, 6288–6308 (2010).

25. Wani, A. *et al.* Surface PEGylation of mesoporous silica nanorods (MSNR): Effect on loading, release, and delivery of mitoxantrone in hypoxic cancer cells. *Sci. Rep.* **7**, 1–11 (2017).

26. Suk, J. S., Xu, Q., Kim, N., Hanes, J. & Ensign, L. M. PEGylation as a strategy for improving nanoparticle-based drug and gene delivery. *Adv. Drug Deliv. Rev.* **99**, 28–51 (2016).

27. Desai, D. *et al.* Targeted modulation of cell differentiation in distinct regions of the gastrointestinal tract via oral administration of differently PEG-PEI functionalized mesoporous silica nanoparticles. *Int. J. Nanomedicine* **11**, 299–313 (2016).

28. He, Q., Zhang, Z., Gao, F., Li, Y. & Shi, J. *In vivo* biodistribution and urinary excretion of mesoporous silica nanoparticles: effects of particle size and PEGylation. *Small* **7**, 271–280 (2011).

29. Asefa, T. & Tao, Z. Biocompatibility of mesoporous silica nanoparticles. *Chem. Res. Toxicol.* **25** 2265–2284 (2012).

30. Jiang, W. *et al.* CuS@MOF-based well-designed quercetin delivery system for chemo-photothermal therapy. *ACS Appl. Mater. Interfaces* **10**, 34513–34523 (2018).

31. Song, Y. *et al.* Selection of DNA aptamers against epithelial cell adhesion molecule for cancer cell imaging and circulating tumor cell capture. *Anal. Chem.* **85**, 4141–4149 (2013).

32. Gao, Y. *et al.* A novel nanomissile targeting two biomarkers and accurately bombing CTCs with doxorubicin. *Nanoscale* **9**, 5624–5640 (2017).

33. Vasanthakumar, S. *et al.* EpCAM as a novel therapeutic target for hepatocellular carcinoma. *J. Oncol. Sci.* **3**, 71–76 (2017).

34. Subramanian, N. *et al.* EpCAM aptamer mediated cancer cell specific delivery of EpCAM siRNA using polymeric nanocomplex. *J. Biomed. Sci.* **22**, 1–10 (2015).

35. Mashreghi, M., Zamani, P., Moosavian, S. A. & Jaafari, M. R. Anti-EpCAM aptamer (Syl3c)-functionalized liposome for targeted delivery of doxorubicin: *In vitro* and *in vivo* antitumor studies in mice bearing C26 colon carcinoma. *Nanoscale Res. Lett.* **15**, (2020).

36. Xie, X. *et al.* EpCAM aptamer-functionalized mesoporous silica nanoparticles for efficient colon cancer cell-targeted drug delivery. *Eur. J. Pharm. Sci.* **83**, 28–35 (2016).

37. Li, Y. *et al.* EpCAM aptamer-functionalized polydopamine-coated mesoporous silica nanoparticles loaded with DM1 for targeted therapy in colorectal cancer. *Int. J. Nanomedicine* **12**, 6239–6257 (2017).

38. Fu, Z. & Xiang, J. Aptamer-functionalized nanoparticles in targeted delivery and cancer therapy. *Int. J. Mol. Sci.* **21**, 1–39 (2020).

39. Iversen, T.-G., Skotland, T. & Sandvig, K. Endocytosis and intracellular transport of nanoparticles: Present knowledge and need for future studies. *Nano Today* **6**, 176–185 (2011).

40. Jalalian, S. H., Ramezani, M., Abnous, K. & Taghdisi, S. M. Targeted co-delivery of epirubicin and NAS-24 aptamer to cancer cells using selenium nanoparticles for enhancing tumor response *in vitro* and *in vivo*. *Cancer Lett.* **416**, 87–93 (2018).

41. Vilotte, F., Jumeau, R. & Bourhis, J. High Z nanoparticles and radiotherapy: a critical view. *The Lancet. Oncology* vol. 20 e557 (2019).

42. Sancey, L. *et al.* The use of theranostic gadolinium-based nanoprobes to improve radiotherapy efficacy. *Br. J. Radiol.* **87**, 1–15 (2014).

43. Dorsey, J. F. *et al.* Gold nanoparticles in radiation research: potential applications for imaging and radiosensitization. *Transl. Cancer Res.* **2**, 280–291 (2013).

44. Chen, Y., Yang, J., Fu, S. & Wu, J. Gold nanoparticles as radiosensitizers in cancer radiotherapy. *Int. J. Nanomedicine* **15**, 9407–9430 (2020).

45. Dou, Y. *et al.* Size-tuning ionization to optimize gold nanoparticles for simultaneous enhanced CT imaging and radiotherapy. *ACS Nano* **10**, 2536–2548 (2016).

46. Subiel, A., Ashmore, R. & Schettino, G. Standards and methodologies for characterizing radiobiological impact of high-Z nanoparticles. *Theranostics* **6**, 1651–1671 (2016).

47. Coulter, J. A. *et al.* Cell type-dependent uptake, localization, and cytotoxicity of 1.9 nm gold nanoparticles. *Int. J. Nanomedicine* **7**, 2673–2685 (2012).

48. Cole, L. E., Ross, R. D., Tilley, J. M., Vargo-Gogola, T. & Roeder, R. K. Gold nanoparticles as contrast agents in X-ray imaging and computed tomography. *Nanomedicine* **10**, 321–341 (2015).

49. Luo, D., Wang, X., Burda, C. & Basilion, J. P. Recent development of gold nanoparticles as contrast agents for cancer diagnosis. *Cancers* **13**, (2021).

50. Xu, W. *et al.* RGD-conjugated gold nanorods induce radiosensitization in melanoma cancer cells by downregulating αvβ3 expression. *Int. J. Nanomedicine* **7**, 915–924 (2012).

51. Zhang, X., Wang, H., Coulter, J. A. & Yang, R. Octaarginine-modified gold nanoparticles enhance the radiosensitivity of human colorectal cancer cell line LS180 to megavoltage radiation. *Int. J.* *Nanomedicine* **13**, 3541–3552 (2018).

52. Karimi, M., Mirshekari, H., Aliakbari, M., Sahandi-zangabad, P. & Hamblin, M. R. Smart mesoporous silica nanoparticles for controlled-release drug delivery. *Nanotechnol. Rev.* **5**, 195–207 (2016).

53. Chang, M. Y. *et al.* Increased apoptotic potential and dose-enhancing effect of gold nanoparticles in combination with single-dose clinical electron beams on tumor-bearing mice. *Cancer Sci.* **99**, 1479–1484 (2008).

54. Shrestha, S., Cooper, L. N., Andreev, O. A., Reshetnyak, Y. K. & Antosh, M. P. Gold nanoparticles for radiation enhancement *in vivo*. *Jacobs J. Radiat. Oncol.* **3**, 139–148 (2016).

55. Liu, S. *et al.* Radiosensitizing effects of different size bovine serum albuminlated gold nanoparticles on H22 hepatoma-bearing mice. *Nanomedicine* **13**, 1371–1383 (2018).

56. Silva, B. L. da *et al.* Magnetic and highly luminescent heterostructures of Gd^3+^/ZnO conjugated to GCIS/ZnS quantum dots for multimodal imaging. *Nanomaterials* **11**, (2021).

57. Li, Z. *et al.* Facile synthesis of Gd-doped CdTe quantum dots with optimized properties for optical/MR multimodal imaging. *J. Biol. Inorg. Chem.* **22**, 1151–1163 (2017).

58. Yu, Y., Yang, T. & Sun, T. New insights into the synthesis, toxicity and applications of gold nanoparticles in CT imaging and treatment of cancer. *Nanomedicine* **15**, 1127–1145 (2020).

59. Bouché, M. *et al.* Recent advances in molecular imaging with gold nanoparticles. *Bioconjug. Chem.* **31**, 303–314 (2020).

60. Liu, Z. *et al.* Virus-inspired hollow mesoporous gadolinium-bismuth nanotheranostics for magnetic resonance imaging-guided synergistic photodynamic-radiotherapy. *Adv. Healthc. Mater.* **11**, 1–16 (2022).

61. Yu, M. & Zheng, J. Clearance pathways and tumor targeting of imaging nanoparticles. *ACS Nano* **9**, 6655–6674 (2015).

62. Kempen, P. J. *et al.* Theranostic mesoporous silica nanoparticles biodegrade after pro-survival drug delivery and ultrasound/magnetic resonance imaging of stem cells. *Theranostics* **5**, 631 (2015).

63. Poon, W. *et al.* Elimination Pathways of Nanoparticles. *ACS Nano* **13**, 5785–5798 (2019).
