# Supplementary material for: Hybridized quantum dot, silica, and gold nanoparticles for targeted chemo-radiotherapy in colorectal cancer theranostics
Source: Commun Biol. 2024 Apr 1;7:393. doi: 10.1038/s42003-024-06043-6 (PMC10984983; doi:10.1038/s42003-024-06043-6)
Supplement: Supplementary file 3 — Description of Additional Supplementary Files [file 42003_2024_6043_MOESM3_ESM.pdf]

Description of Additional Supplementary Files

File name: Supplementary data 1
Description: All the source data behind the graphs in the paper
